# Agreements, Behaviour, and Change: Sex Outside the Relationship in Male HIV-negative Partners in HIV Serodiscordant Relationships in Australia, Brazil, and Thailand

**DOI:** 10.1007/s10461-023-04030-2

**Published:** 2023-03-14

**Authors:** James Gray, Garrett Prestage, Fengyi Jin, Nittaya Phanuphak, Ruth K. Friedman, Christopher K Fairley, Anthony Kelleher, David J Templeton, Iryna Zablotska-Manos, Jennifer Hoy, Anna McNulty, David Baker, Graham Brown, Andrew Grulich, Benjamin Bavinton, for the Opposites Attract study group

**Affiliations:** 1 The Kirby Institute, UNSW Sydney, Sydney, NSW 2052 Australia; 2 Institute of HIV Research and Innovation, Bangkok, Thailand; 3 Instituto Nacional de Infectologia Evandro Chagas, Fundação Oswaldo Cruz, Rio de Janeiro, Brazil; 4 Melbourne Sexual Health Centre, Melbourne, Australia; 5 Central Clinical School, Monash University, Melbourne, Australia; 6 Department of Sexual Health Medicine and Sexual Assault Medical Service, Sydney Local Health District, Camperdown, Australia; 7 Discipline of Medicine, Central Clinical School, Faculty of Medicine and Health, The University of Sydney, Sydney, Australia; 8 Westmead Clinical School, Faculty of Medicine and Health, University of Sydney, Sydney, Australia; 9 Western Sydney Sexual Health, Western Sydney Local Health District, Sydney, Australia; 10 Department of Infectious Diseases, Alfred Hospital, Monash University, Melbourne, Australia; 11 Sydney Sexual Health Centre, Sydney and Sydney Eye Hospital, Sydney, Australia; 12 School of Population Health, UNSW Sydney, Sydney, Australia; 13 East Sydney Doctors, Sydney, Australia; 14 Centre for Social Impact, UNSW Sydney, Sydney, Australia

**Keywords:** Serodiscordant couples, Homosexual, Gay men, Men who have sex with men, HIV prevention, Risk reduction strategies, Sexual behavior

## Abstract

Male HIV serodiscordant couples have diverse relationship agreements regarding sex outside the relationship. We examined the relationship agreements as described by 343 male HIV-negative partners in HIV serodiscordant relationships in Australia, Brazil and Thailand participating in a multi-year cohort study. At baseline, 125 (34.1%) HIV-negative partners reported no agreement, 115 (33.5%) had a monogamous agreement, and 103 (37.9%) had an open agreement allowing sex outside the relationship. Relationship agreements were largely stable over time, with 76% of HIV-negative men reporting the same agreement across follow up, while changes were predominantly towards having an open agreement. Behaviour largely matched relationship agreements, and the predictors of breaking an agreement by having condomless anal intercourse (CLAI) with an outside partner were CLAI within the relationship (OR = 3.17, 95%CI: 1.64–6.14, p < 0.001) and PrEP use in the last three months (OR = 3.42, 95%CI: 1.48–7.92, p = 0.004). When considering HIV transmission risk for HIV-negative men in serodiscordant relationships, greater focus needs to be placed on sex that is occurring outside the relationship and the agreements that facilitate this.

## Introduction

The last decade has seen substantial changes in HIV prevention and care due to improved understanding of the benefits of early treatment for both the health of the individual living with HIV [1] and the prevention of onward HIV transmission [2–4]. HIV serodiscordant couples have been seen as an important context of HIV transmission [5, 6] as anal sex presents a high risk of HIV transmission [7] in the absence of protective behaviour. However, increasing access to treatment [8], community education about undetectable viral load (VL) in the context of U = U [9], and the introduction of HIV pre-exposure prophylaxis (PrEP) [10] have presented new ways for HIV serodiscordant couples to effectively eliminate HIV transmission within the context of their relationships, beyond condom use. This includes the couple using knowledge of the HIV-positive partner’s viral load to make agreements about condomless anal intercourse (CLAI) within the relationship [11, 12].

Male couples have diverse types of relationships, and it is common to have additional sexual partners outside of the primary relationship [13–17]. This behaviour may be regulated through the use of explicit, spoken relationship agreements which may take multiple forms, and is distinct from sexual arrangements which relate to implicit understandings of what is allowed in the relationship [18]. Where couples do agree that sex can occur with other partners, agreements can cover sexual activity that may occur together, apart, or both. Sex outside the relationship can also occur in the absence of an agreement [19–22]. The agreement about other sexual partners may also change, often beginning with an initial period of sexual exclusivity and moving to a relationship that includes other sexual partners [21–24].

In some communities, a well-documented behavioural approach for HIV-negative seroconcordant couples to prevent HIV transmission within the relationship is called negotiated safety. This involves explicitly negotiating forms of sexual behaviour in the relationship, including an agreement for monogamy, limiting sex with outside partners to behaviours that are low risk for HIV such as oral sex, or having an agreement to only have CLAI with their primary partner and to use condoms for anal sex outside the relationship [25–27]. The desire to engage in CLAI has been associated with trust and intimacy within a relationship and that it typically happens early in the relationship [28]. However, the presence of a negotiated safety agreement does not guarantee compliance and risky CLAI occur can still occur [29]. More generally, agreements do not always match behaviours and agreements may be broken for a range of reasons with HIV serodiscordant couples reporting different reasoning compared to seroconcordant couples, including potential concerns about HIV transmission [30].

Studies in Australia demonstrate between 40.0% and 53.8% of gay and bisexual men living with HIV are in serodiscordant relationships, while between 3.7% and 5.7% of HIV-negative gay men are in a serodiscordant relationship [13, 14, 31]. Prior to studies showing that HIV treatment leading to undetectable VL eliminated HIV transmission risk, the HIV-negative partner in serodiscordant relationships was encouraged to use condoms with all partners [32]. In the context of HIV serodiscordant couples, there is currently no similar, standardised advice about effective negotiation of CLAI in the presence of undetectable VL [11, 12]. The role of PrEP in developing relationship agreements has yet to be the focus of widespread health promotion responses [33] and potentially affecting how sex can be enacted with outside partners [34].

There are gaps in the literature about the agreements that HIV serodiscordant couple make regarding sex outside the relationship. First, research in this area has predominantly been conducted in North American and Western Europe, with very little research available from Asia or South America. Second, for HIV serodiscordant couples where the HIV positive partner has an undetectable VL, HIV transmission risk primarily comes from external sexual partners, and this highlights the importance of understanding sexual agreements and what happens when they are broken. Finally, the mechanics of how U = U is operationalised in the context of serodiscordant couples is not well described. The aims of this analysis were to: (1) describe the characteristics of couples where the HIV-negative partner reported different relationships agreements; (2) explore factors that predict having agreements allowing sex outside the relationship; (3) describe the ways that the HIV-negative partner reported the relationship agreements were broken; (4) explore the predictors of breaking agreements by having CLAI outside the relationship; and (5) describe how these agreements for sex outside the relationship changed over time.

## Methods

### Participants

Participants were gay and bisexual men in the Opposites Attract study for which the design and methods have previously been published [35]. Briefly, data were collected as part of a prospective, observational cohort study of sexually active HIV serodiscordant male couples recruited through high HIV caseload clinics and hospitals in Australia, Brazil, and Thailand. The primary goal of the study was to determine the impact of antiretroviral therapy (ART) on the prevention of HIV transmission among gay and bisexual men.

To be eligible to participate, both men in the couple, defined here as study partners, had to be at least 18 years old, one partner be HIV-positive and the other partner HIV-negative at baseline, be having anal sex at least once a month on average, and agree to attend clinic visits at least twice a year. Enrolment occurred between 2012 and 2014, with follow up through to the end of 2016. Couples were followed up until the end of the study (n = 230) or they withdrew or were lost to follow up (n = 41) or until they became ineligible (n = 72). Among those that became ineligible, 60 (83%) ceased within-couple anal intercourse entirely or broke-up, ten (14%) reported anal intercourse less than once per month on average, and two (3%) died in separate couples.

### Procedures

Couples were required to attend at least two clinic visits each year and behavioural and attitudinal information was collected through online computer-assisted self-interview questionnaires conducted at the time of each study visit.

The questionnaires for the HIV-positive and HIV-negative partners were different, with less detail about sexual acts asked of the HIV-positive partners due to public health legislation in place at the time in Australia. The questionnaires were available in English, Brazilian Portuguese, and Thai. Clinical data were collected via electronic case report forms and included ART regimen and viral load of the positive partner, HIV antibody test results for the negative partner, and STI test results for both partners. Ethics approvals were obtained in all three countries.

### Measures

Demographic characteristics including age, education and ethnicity were collected from both partners, as well as information about the length of time since they first had sex with each other and whether they lived together. Both partners were asked about whether they had sexual contact with any men other than their study partner, and if so, whether these other partners were regular, how many they had, and whether they were believed to be HIV-positive or HIV-negative.

The HIV-negative partner was asked about their explicit, spoken agreements with their study partner about sex with other men, including what this involved (may not have sex at all, may not have anal sex, may only have sex with condoms, may have sex without condoms, other). The HIV-negative partner was asked about sexual behaviour with others in the previous 3 months, whether they themselves had anal intercourse with other men, and how many times they had anal intercourse with other partners (never, once, twice, 3–5 times, 6–10 times, 11–30 times, 31–50 times, over 50 times) for both insertive and receptive anal intercourse. These questions were asked by perceived HIV VL status of the partners (undetectable, low, moderate, high, he has not received the results yet, I don’t know), and for anal intercourse with condoms, CLAI without ejaculation, and CLAI with ejaculation. The HIV-negative partners were also asked whether they believed their HIV-positive study partner had sex with other men.

For this analysis, we defined a “monogamous agreement” as an explicit, spoken agreement between the two study partners that neither partner was permitted to have sex with other partners, either together or separately. The HIV-negative partners were asked about condomless anal intercourse within their regular partner (CLAIR) and condomless anal intercourse with casual partners (CLAIC). We defined “open agreements disallowing CLAIC” as any agreement allowing sex with other partners but not permitting CLAIC with other partners, and “open agreements allowing CLAIC” as any agreements explicitly allowing sex with other partners that could include CLAIC. A “broken agreement with CLAIC” was defined as sexual behaviour with other partners in the presence of a monogamous agreement, or CLAIC with other partners in the presence of an open agreement disallowing CLAIC.

Several key variables were constructed from the items above. The variable of open agreement disallowing CLAIC included couples that indicated they could have sex with others (may not have anal sex, may only have sex with condoms) but not CLAIC. As agreements could change over time, variables that explored discrepancies between agreements and behaviour were constructed so that the behaviour was matched to times when specific agreements were in place.

### Analysis

Data were analysed using Stata 15.1 (Stata Corporation, College Station, Texas, USA). Couples were excluded if they attended the baseline visit but had no follow up visits (n = 13). Where couples became ineligible (n = 72), data collected prior to them becoming ineligible was retained in the analysis. Descriptive analyses of the agreement variables were conducted, including the examination of country differences, using Pearson’s chi-squared tests. Baseline associations between a range of variables and the different relationship agreement types were examined using Pearson’s chi-square tests.

Generalised linear models were used to examine associations over follow-up between a range of variables and having an agreement for sex outside the relationship as reported by the HIV-negative partner. A generalised linear model was also used to examine associations with breaking a monogamous or open agreement disallowing CLAIC, by having CLAIC with an external partner. The strengths of the associations were presented as odds ratios (OR) or adjusted odds ratios (aOR), and their corresponding 95% confidence intervals (95%CI), and p-values. These analyses were repeated for each of the three countries to check for differences by country.

Incidence rates of change to different relationship types were calculated by dividing the number of couples who changed to a particular agreement type by the couple-years of follow-up; we reported the incidence rate (IR) and 95%CI.

## Results

At baseline, 125 (34.1%) HIV-negative men reported no agreement, 115 (33.5%) had a monogamous agreement, and 103 (37.9%) had an open agreement allowing sex outside the relationship. Of those with any open agreement (both with and without CLAIC), 84 HIV-negative men (81.6% of those with any open agreement, 24.5% of all couples) had an open agreement disallowing CLAIC and 19 (18.4% of those with any open agreement, 5.5% of all couples) had an open agreement allowing CLAIC. There were substantial differences by country: 19.6% of Australian HIV-negative men reported no agreement, compared to 50.5% of Brazilian and 49.5% of Thai; 41.9% of Australian HIV-negative men had a monogamous agreement, compared to 34.4% of Brazilian and 19.6% of Thai; 38.6% of Australian HIV-negative men had an open agreement disallowing CLAIC, compared to 14.0% of Brazilian and 30.9% of Thai; and 11.1% of Australian HIV-negative men had an open agreement allowing CLAIC, compared to 1.1% of Brazilian and 1.0% of Thai (χ^2^ = 51.666, p < 0.001).

There were significant baseline associations with the four agreement types (no agreement, monogamous agreement, open agreement disallowing CLAIC, and open agreement allowing CLAIC) and a number of covariates (Table 1). There were significant differences in relationship type based on length of relationship (χ^2^ = 13.308, p = 0.008). Not having an agreement became less common as relationship length increased (less than 12 months: 41.6%, one to five years: 38.4%, more than five years: 20.0%) and monogamous agreements were much more common in men in the first year of their relationship (less than 12 months: 53.0%, one to five years: 29.6%, more than five years: 17.4%). There was an increase in agreements to have sex with other men without CLAIC as time increased since the couple first had sex (less than 12 months: 28.6%, one to five years: 34.5%, more than five years: 36.9%). Among HIV-negative men that had an open agreement allowing CLAIC it was less common to have this agreement as relationship length increased (less than 12 months: 42.1%, one to five years: 36.8%, more than five years: 21.1%). Significant differences in CLAIR (χ^2^ = 16.208, p = 0.001) was reported with HIV-negative men that had a monogamous agreement (61.7% vs. 38.3%) and HIV-negative men with an open agreement allowing CLAIC (89.5% vs. 10.5%). However, a lower proportion of CLAIR was reported by HIV-negative men with no agreement (46.4% vs. 53.6%) or open agreement disallowing CLAIC (51.2% vs. 48.8%). There were significant differences in the reported perceived VL of the HIV-positive partner. A lower proportion of HIV negative men with no agreement (47.2% vs. 52.8%) reported that they perceived their partners to have an undetectable VL, while higher proportion reported this when they had a monogamous agreement (61.7% vs. 38.3%) or an open agreement allowing CLAIC (76.7% vs. 26.3%). Those with an open agreement disallowing reported no difference (50.0% vs. 50.0%). Differences in the reported agreements to have CLAIR (p < 0.001). A lower proportion of agreements for CLAIR was reported by HIV negative men with no agreement (22.9% vs. 77.1%), a monogamous agreement (40.0% vs. 60.0%) or an open agreement disallowing CLAIC (33.3% vs. 66.7%). Those with an open agreement allowing CLAIC reported a higher proportion (84.6% vs. 15.4%).

**Table 1:** Baseline characteristics of couples that had no agreement about sex with outside partners, monogamous agreements, open agreements disallowing CLAI, and open agreements allowing CLAIC

	No agreement	Monogamous agreement	Open agreement disallowing CLAIC	Open agreement allowing CLAIC	χ^2^, p-value*
	n/N (%)	n/N (%)	n/N (%)	n/N (%)	
Country					
Australia Brazil Thailand	30/125 (24.0) 47/125 (37.6) 48/125 (38.4)	64/115 (55.7) 32/115 (27.8) 19/115 (16.5)	42/84 (50.0) 13/84 (15.5) 30/84 (34.5)	17/19 (89.5) 1/19 (5.2) 1/19 (5.2)	**51.666, p < 0.001**
Age of HIV-negative partner					
Under 30 years 30–39 years 40 years and over	50/125 (40.0) 39/125 (31.2) 36/125 (28.8)	35/115 (30.4) 38/115 (33.0) 42/115 (36.5)	24/84 (28.6) 30/84 (35.7) 30/84 (35.7)	4/19 (21.1) 7/19 (36.8) 8/19 (42.1)	5.453, p = 0.487
Education					
High School or less Vocational University	42/125 (33.6) 24/125 (19.2) 59/125 (47.2)	28/115 (24.6) 16/115 (14.0) 70/115 (16.4)	17/84 (20.2) 17/84 (20.2) 50/84 (59.5)	6/19 (31.6) 4/19 (21.1) 9/19 (47.4)	7.989, p = 0.239
Employed full-time					
No Yes	61/125 (48.8) 64/125 (51.2)	47/115 (40.9) 68/115 (59.1)	26/84 (31.0) 58/84 (69.1)	7/19 (36.8) 12/19 (63.2)	6.779, p = 0.079
Living together full-time					
No Yes	49/125 (39.2) 76/125 (60.8)	43/115 (37.4) 72/115 (62.6)	24/84 (28.6) 60/84 (71.4)	6/19 (31.6) 13/19 (68.4)	2.813, p = 0.421
First sex within the couple					
Less than 12 months 1–5 years 5 or more years	52/125 (41.6) 48/125 (38.4) 25/125 (20.0)	61/115 (53.0) 34/115 (29.6) 20/115 (17.4)	24/84 (28.6) 29/84 (34.5) 31/84 (36.9)	8/19 (42.1) 7/19 (36.8) 4/19 (21.1)	**17.308, p = 0.008**
Having CLAI with partner					
No Yes	67/125 (53.6) 58/125 (46.4)	44/115 (38.3) 71/115 (61.7)	43/84 (51.2) 41/84 (48.8)	2/19 (10.5) 17/19 (89.5)	**16.208, p = 0.001**
Perceived VL of study partner					
Undetectable Detectable/Unknown	59/125 (47.2) 66/125 (52.8)	74/115 (64.4) 41/115 (35.7)	42/84 (50.0) 42/84 (50.0)	14/19 (73.7) 5/19 (26.3)	**10.664, p= 0.014**
Have agreements allowing CLAI inside the couple					
No Yes	64/125 (77.1) 19/125 (22.9)	57/115 (60.0) 38/115 (40.0)	46/84 (66.7) 23/84 (33.3)	2/19 (15.4) 11/19 (84.6)	**20.544, p < 0.001**
PrEP use in previous 3 months					
No Yes	115/125 (92.0) 10/125 (8.0)	101/115 (87.8) 14/115 (12.2)	76/84 (90.5) 8/84 (9.5)	16/19 (84.2) 3/19 (15.8)	1.839, p = 0.606

*p-values are test of difference

Table 2 out lines factors associated with having each agreement type at any point over follow-up using multivariate generalised linear models. For no agreements, the only significant difference was by country with Brazilian men (aOR = 7.28, 95%CI: 2.93–18.14, p < 0.001) and Thai men (aOR = 4.58, 95%CI: 1.50-13.98, p = 0.006) both more likely to have no agreement. For HIV-negative men reporting a monogamous agreement, this was associated with living together full-time (aOR = 2.15, 95%CI: 1.14–4.03, p = 0.017), and with decreasing time since first sex (1–5 years: aOR = 0.43, 95%CI: 0.21–0.87, p = 0.019; 5 or more years: aOR = 0.35, 95%CI: 0.15–0.84, p = 0.018). Having an open agreement disallowing CLAI was associated with having an agreement to have CLAI inside the relationship. Finally, having an open agreement allowing CLAI was associated perceiving their study partner to have an undetectable VL (aOR = 3.68, 95%CI: 1.17–11.55, p = 0.026), having an agreement to have CLAI inside the relationship (aOR = 18.94, 95%CI: 7.23–49.64, p < 0.001) and not living together full time (aOR = 0.32, 95%CI: 0.11–0.96, p = 0.042). When combining the two open relationship categories, HIV-negative partners that had used PrEP in the last three months were more likely to have an open agreement allowing CLAIC (aOR = 2.02, 95%CI: 1.10–3.73, p = 0.023).

**Table 2:** Multivariate generalised linear models of variables associated with having different agreement types for sex outside the relationship over follow up

	No Agreement (aOR, 95%CI)	p-value	Monogamy (aOR, 95%CI)	p-value	No CLAIC (aOR, 95%CI)	p-value	CLAIC Allowed (aOR, 95%CI)	p-value
Country								
Australia Brazil Thailand	1 **7.28 (2.93–18.14)** **4.58 (1.50-13.98)**	**p < 0.001** **p = 0.006**	1 0.72 (0.35–1.50) 0.56 (0.23–1.35)	p = 0.386 p = 0.196	1 0.40 (0.15–1.10) 0.59 (0.21–1.66)	p = 0.077 p = 0.322	1 0.21 (0.03–1.40) 0.47 (0.09–2.38)	p = 0.107 p = 0.360
Age of HIV-negative partner								
Under 30 years 30–39 years 40 years and over	1 1.42 (0.62–3.27) 1.24 (0.58–2.68)	p = 0.400 p = 0.664	1 1.09 (0.49–2.44) 0.90 (0.39–2.10)	p = 0.825 p = 0.812	1 1.05 (0.45–2.44) 1.23 (0.49–3.10)	p = 0.914 p = 0.653	1 1.27 (0.39–4.14) 1.04 (0.31–3.48)	p = 0.693 p = 0.953
Education								
High School or less Vocational University	1 2.36 (0.93–5.96) 1.01 (0.46–2.19)	p = 0.071 p = 0.984	1 0.47 (0.16–1.36) 1.27 (062-2.64)	p = 0.166 p = 0.514	1 2.36 (0.77–7.23) 1.14 (0.46–2.85)	p = 0.131 p = 0.778	1 0.62 (0.15–2.56) 0.48 (0.15–1.55)	p = 0.511 p = 0.219
Employed full-time	0.68 (0.32–1.47)	p = 0.329	0.61 (0.32–1.19)	p = 0.148	1.53 (0.71–3.29)	p = 0.272	3.08 (0.89–10.59)	p = 0.075
Live Together full-time								
No Yes	1 0.65 (0.35–1.22)	p = 0.182	1 **2.15 (1.14–4.03)**	**p = 0.017**	1 0.77 (0.39–1.51)	p = 0.445	1 **0.32 (0.11–0.96)**	**p = 0.042**
First sex within the couple								
Less than 12 months 1–5 years 5 or more years	1 1.80 (0.88–3.65) 1.03 (0.41–2.61)	p = 0.104 p = 0.964	1 **0.43 (0.21–0.87)** **0.35 (0.15–0.84)**	**p = 0.019** **p = 0.018**	1 0.84 (0.36–1.97) 1.59 (0.60–4.23)	p = 0.685 p = 0.356	1 1.31 (0.45–3.80) 0.87 (0.26–2.91)	p = 0.622 p = 0.827
Have CLAI together	1.88 (0.90–3.94)	p = 0.095	0.85 (047-1.56)	p = 0.608	1.02 (0.51–2.04)	p = 0.950	1.01 (0.49–2.08)	p = 0.968
Perceived study partner to have detectable VL	0.74 (0.36–1.55)	p = 0.431	0.62 (0.34–1.14)	p = 0.124	0.90 (0.43–1.88)	p = 0.784	**3.68 (1.17–11.55)**	**p = 0.026**
Agreement to have CLAI inside relationship	0.55 (0.21–1.44)	p = 0.223	1.72 (0.81–3.64)	p = 0.155	**0.35 (0.13–0.92)**	**p = 0.033**	**18.94 (7.23–49.64)**	**p < 0.001**
PrEP use in previous 3 months								
No Yes	0.50 (0.24–1.08)	p = 0.078	0.71 (0.37–1.37)	p = 0.308	1.68 (0.88–3.21)	p = 0.117	2.02 (0.93–4.37)	p = 0.075

Over follow up, there were differences between agreements and the HIV-negative partner’s reported behaviour (Table 3). There were 62.6% of HIV-negative men that had no agreement at any time during follow up. Within this group, 74.8% of HIV-negative partners reported at least one period when they did not have sex with another partner, 42.2% had sex with other partners with condoms at least once, and 32.6% had CLAIC at least once. Of the 51.3% HIV-negative men that reported a monogamous agreement at some point over follow up, 85.9% of HIV-negative partners reported at least one period when they did not have sex with another partner, 26.6% had sex with other partners with condoms at least once, and 22.7% had CLAIC at least once. During follow up, 44.9% of HIV-negative men had an open agreement disallowing CLAIC at some point. Of these, 35.8% of HIV-negative partners reported at least one period when they did not have sex with another partner, 58.7% had sex with other partners with condoms at least once, and 38.5% had CLAIC at least once. Finally, there were 15.5% of HIV-negative men that had an open agreement allowing CLAI at some point over follow up. Of these, 24.3% of HIV-negative partners reported at least one period when they did not have sex with another partner, 18.9% had sex with other partners with condoms at least once, and 72.0% had CLAIC at least once.

**Table 3:** Alignment between agreements and HIV-negative partner’s reported behaviour in couples that had no agreement regarding sex outside, couples that were monogamous and those that had agreements for sex outside, across follow up

	Did not engage in sex with other partners	Had sex with other partners with condoms	Had CLAI with other partners
**No agreement**	101/135 (74.8%)	57/135 (42.2%)	44/135 (32.6%)
**Monogamous agreement**	110/128 (85.9%)	34/128 (26.6%)	29/128 (22.7%)
**Open agreement disallowing CLAI**	39/109 (35.8%)	64/109 (58.7%)	42/109 (38.5%)
**Open agreement allowing CLAI**	9/37 (24.3%)	7/37 (18.9%)	27/37 (72.0%)

The factors associated with breaking an agreement by having CLAIC (Table 4) were engaging in CLAIR with the study partner (aOR = 3.17, 95%CI: 1.64–6.14, p = 0.001) and the HIV-negative partner being on PrEP in the last three months (aOR = 3.42, 95%CI: 1.48–7.92, p = 0.004).

**Table 4:** Generalised linear models of variables associated with having CLAI when breaking agreements

	Break agreement with CLAI – bivariate (OR, 95%CI)	p-value	Break agreement with CLAI - multivariate (aOR, 95%CI)	p-value
Country				
Australia	1		1	
Brazil	**0.41 (0.18–0.96)**	**p = 0.041**	0.38 (0.08–1.83)	p = 0.227
Thailand	**0.26 (1.23–0.57)**	**p = 0.001**	0.34 (0.08–1.50)	p = 0.115
Age of HIV-negative partner				
Under 30 years	1		1	
30–39 years	1.91 (0.86–4.27)	p = 0.113	1.21 (0.45–3.27)	p = 0.702
40 years and over	1.09 (0.69–3.61)	p = 0.275	0.69 (0.22–2.12)	p = 0.516
Education				
High School or less	1		1	
Vocational	1.72 (0.62–4.77)	p = 0.300	2.29 (0.75–7.01)	p = 0.148
University	0.81 (0.36–1.82)	p = 0.605	0.95 (0.35–2.55)	p = 0.914
Employed full-time	1.27 (0.56–2.89)	p = 0.572	1.08 (0.46–2.54)	p = 0.867
Live Together full-time	1.18 (0.47–2.98)	p = 0.727	0.76 (0.29–1.99)	p = 0.569
First sex within the couple				
Less than 12 months	1		1	
1–5 years	1.33 (0.51–3.50)	p = 0.565	1.01 (0.35–2.89)	p = 0.986
5 or more years	2.17 (0.82–5.72)	p = 0.117	1.56 (0.51–4.80)	p = 0.427
Have CLAI together	**0.36 (0.21–0.63)**	**p < 0.001**	**3.17 (1.64–6.14)**	**p = 0.001**
Perceived study partner to have detectable VL	**4.15 (2.44–7.06)**	**p < 0.001**	0.58 (0.25–1.39)	p = 0.226
Agreement to have CLAI inside relationship	**2.31 (1.02–5.20)**	**p = 0.044**	0.81 (0.33–2.01)	p = 0.653
PrEP use in previous 3 months	**2.90 (1.57–5.36)**	**p = 0.001**	**3.42 (1.48–7.92)**	**p = 0.004**

Over follow-up (Table 5), 268 (78.1%) HIV-negative men reported no change to their relationship agreements, and among this group, 93 (34.7%) never had an agreement about sex outside the relationship, 92 (34.3%) maintained an agreement to not have sex with other partners. Among the HIV-negative men that maintained agreements that allowed sex outside the relationship (n = 79), 67 (84.8%) maintained open agreements disallowing CLAIC, while 12 (15.2%) maintained open agreements allowing CLAIC. Four (1.5%) HIV-negative men maintained ‘other agreements’ which were not otherwise defined.

**Table 5:** Consistency and changes in agreements about sex with other partners, across follow up and stratified by country

	Total n = 352*	Australia n = 158	Brazil n = 95	Thailand n = 99	X2, p-value
Always no agreement	93	18 (19.4%)	39 (41.9%)	36 (38.7%)	**32.441, p < 0.001**
Always monogamous	92	50 (54.4%)	27 (29.4%)	15 (16.3%)	**11.447, p = 0.003**
Always open agreement disallowing CLAI	67	33 (48.5%)	10 (14.7%)	24 (36.8%)	**8.519, p = 0.014**
Always open agreement allowing CLAI	12	10 (83.3%)	1 (8.3%)	1 (8.3%)	**8.379, p = 0.015**
Always other agreement	4	4 (100.0%)	0 (0.0%)	0 (0.0%)	5.402, p = 0.067
To no agreement	18	10 (55.6%)	4 (22.2%)	4 (22.2%)	0.925, p = 0.630
To monogamous	13	5 (38.5%)	3 (23.1%)	5 (38.5%)	0.691, p = 0.708
To open agreement disallowing CLAI	28	9 (32.1%)	9 (32.1%)	10 (35.7%)	1.942, p = 0.379
To open agreement allowing CLAI	19	15 (79.0%)	1 (5.3%)	3 (15.8%)	**9.969, p = 0.007**
To other	6	4 (66.7%)	1 (16.7%)	1 (16.7%)	1.203, p = 0.548

*Seventy-five couples had a total of 84 changes to their agreements

Seventy-five (21.9%) HIV-negative men reported a change in their agreement about sex with men outside the relationship. There were 84 changes in agreements from this group, with 11 HIV-negative men changing agreements twice and one changing three times (Table 5). Eighteen of the 84 (21.4%) changes were to no agreement, 13 (15.5%) changes were to monogamous agreements, 28 (33.3%) changes were to open agreements disallowing CLAI, 19 (22.6%) changes were to open agreement allowing CLAIC, and six (7.1%) were changes to ‘other’ which were not further described. Of the changes to open agreement allowing CLAIC, Australian HIV-negative men comprised 15 (79.0%) of the changes, compared to 6.3% of Brazilian changes and 4.8% of Thai changes (χ^2^ = 9.969, p = 0.007). The incident rate of moving to an open agreement disallowing CLAIC was 4.84 per 100-person years (95% CI = 3.34–7.01). The incident rate of moving to an open agreement allowing CLAIC was 3.31 per 100-person years (95% CI = 2.12–5.18).

## Discussion

In this study of gay and bisexual men in Australia, Brazil and Thailand, explicit, spoken agreements about sex outside the relationship were relatively common, with more than two thirds of HIV-negative partners reporting an agreement in some form. There were significant differences by country, with Australian couples much more likely to have any form of agreement at baseline and these couples continued to have agreements at a higher rate across follow up. Relationship agreements were relatively stable across follow up, with more than three quarters not changing from baseline. The changes that were made were predominantly in the direction of more openness and more CLAIC. Over the course of the study, the proportion of HIV-negative partners reporting sexual behaviour that broke their monogamous agreements increased.

One of the primary prevention tools for HIV-negative men in an open relationship is the use of PrEP. Previously published analysis of the 31,532 sex acts that occurred within the relationship in this study [36] found that 46.7% of acts were protected by condoms, and 48.6% were protected by undetectable viral load and/or PrEP, leaving 4.7% that were protected by neither. The use of PrEP was not significantly associated with the relationship agreement type in multivariate analyses although it becomes significant when combing the two open relationship categories. The decision to utilise PrEP may reflect concern about transmission within the relationship as the effectiveness of treatment as prevention (TasP) in HIV prevention had not been proven in this cohort at the time of data collection. However, it is likely to also be related to providing protection from HIV acquisition from outside the relationship. The importance of PrEP has been found to be higher in ‘open’ and ‘monogamish’ relationships compared to relationships that didn’t allow any other sexual partners [37], where ‘monogamish’ was defined as sex with other partners in limited circumstances that involved both primary partners.

Australian couples were more likely to have any relationship agreement, and this is likely to relate to the promotion of relationship agreements to reduce HIV risk in Australia over several decades [26, 27]. Similarly, the lower levels of relationships agreements for sex with outside partners may relate to the lower presence of relationships agreements of any type in Brazil and Thailand. However, previous research of male couples where behavioural data is available from both members has identified reasonably consistent sexual behaviours outside the relationship, even in the absence of an explicit relationship agreement and this has been termed an implicit agreement [18]. Connected to this, nearly three quarters (74.8%) of those that did not have an agreement, did not have sex with other partners, which may reflect an implicit understanding of what is allowed in the relationship, or alternatively a lack of intent or opportunity to meet other sexual partners. Additionally, at baseline longer term couples were less likely to have an agreement allowing CLAIC, despite being more likely to have an open agreement without CLAIC permitted. This may reflect that this group was on average older, Australian and may have been impacted by direct experiences of the most severe impacts of the HIV epidemic, as well as decades of condom reinforcement.

Across follow up, broken agreements were relatively common, with 26.6% of HIV-negative men who had a monogamous agreement reporting extra-dyadic sex with condoms, and 22.7% reporting CLAIC. Among couples that had an agreement not to have CLAIC, 38.5% HIV-negative men reported CLAIC occurring. Broken relationship agreements have implications for the relationship and for the sexual health of both partners. The health implications for the HIV-positive partner are likely to be related to sexually transmissible infections, while the HIV-negative partner could contract HIV from outside partners [3]. If the couple was having CLAIR within their relationship the HIV-negative partner was more likely to have CLAIC, regardless of what their relationship agreement said. This may reflect that the behaviour has been normalized within the relationship and the HIV-negative partner was seeking similar sex outside. However, when the behaviour was contrary to their agreement, particularly for those with monogamous agreements, there was the potential to undermine the trust and commitment of their relationship [38] and they may hide what they are doing and be less likely to seek support in the form of testing, access to non-occupational HIV post-exposure prophylaxis (nPEP) or PrEP. Previous research has also shown that the partners in seroconcordant and serodiscordant couples can have different reasons for breaking their agreements [30] and these differences revolved around the type of sex that was felt to be safe at the time. As HIV prevention has evolved to include TasP and PrEP, those differences between HIV seroconcordant and HIV serodiscordant couples may reduce. This study did not investigate the impact of alcohol and other drugs on relationship agreements and behaviour. The use of drugs connected with the gay club scene has been found to predict the frequency and occurrence of casual CLAI for people with non-monogamous agreements in particular, but also for those with monogamous agreements [39].

An important difference in the multivariate analysis was the different associations between having an agreement to have CLAI in the relationship and the different relationship types. Having CLAI inside the relationship was associated with having both types of open relationship agreement, but not with monogamy. Conceptually a monogamous relationship should be safer for CLAI and therefore it could be expected that this an agreement to have CLAI would be associated with monogamy. However, a number of factors may work against this. First, this study occurred as evidence was emerging for U = U [3, 4] and in the context of sero-discordant relationships it is likely that there was a diversity of understanding about the impact of treatment as prevention. Additionally, it may also reflect a lack of trust that the monogamous behaviour will continue as across follow up in this study, only 63.6% of HIV-negative men reported that they behaved consistently with their monogamous agreement.

This analysis raises a number of implications for further health promotion, education, and research for male HIV serodiscordant couples. Compared to not having an agreement, an agreement for monogamy reduced the proportion of respondents reporting CLAIC, which having an open relationship, whether CLAIC was allowed or not, increased the proportion of CLAIC occurring. The potential for the HIV-negative partner to attempt to use the skills they have developed discussing VL within the relationship to underpin their approach to negotiating condomless sex outside the relationship should be addressed. Prior to supressed VL being demonstrated to virtually eliminate HIV transmission risk in gay male couples, CLAIC was found to be more common when VL was discussed [40]. While the underpinning principles of TasP continue to apply, the ability to negotiate the use of undetectable VL for CLAIC may be more difficult and less accurate. There is the potential for HIV-negative partners in serodiscordant couples when the HIV-positive partner is on ART to not engage with health promotion programs targeted towards HIV prevention, as it may not speak to their specific circumstances and risks of HIV acquisition from sex outside the relationship. Furthermore, there are opportunities for this to align with greater promotion of U = U, both publicly and between service providers and their clients. This promotion should be tailored to build the knowledge of community members, regardless of their HIV status, and of the services that work with them. Consideration of the evolution of negotiated safety [25–27] could provide a guide to this work. The behaviours associated with negotiated safety were developed in the community in response to a changing understanding of HIV and when it was subsequently described in the literature, this enabled communities, healthcare professionals and organisations to work together to strengthen and enhance its use. Finally, there is a large body of research on relationship agreements in the Australian literature, however there are opportunities for research on the impact of the Brazilian and Thai cultural contexts regarding relationship agreements and negotiation.

These analyses had several limitations. Data collection began prior to the impact of TasP being confirmed and continued as this was communicated to communities, and this may have had an impact on the negotiation of open relationships over the course of the study. While this was a relatively large sample in the context of serodiscordant couples, the sample size led to large confidence intervals and a larger sample may have uncovered additional associations. The differences in the number of couples recruited in Australia compared to Brazil and Thailand meant that factors where there was an association in Australia but not the other countries may reflect the difference in statistical power. Questions about relationship agreements and about the type of sex that was occurring were only asked of the HIV-negative study partner due to the potential prosecution for HIV transmission of HIV-positive partners in cases of phylogenetically linked HIV transmission. The future exploration of HIV-positive partner perspectives has the potential to reveal further nuance about the way agreements are created and enacted. By asking about relationship agreements in the questionnaires, we may have prompted these discussions over the course of the study. The participating couples may not be representative of male HIV serodiscordant couples in the three countries, as they were drawn from clinics in urban areas and were therefore more likely to be connected to care.

## Conclusion

This study adds further weight to the idea that the risk of HIV transmission within HIV-serodiscordant couples has significantly reduced due to impact of TasP and PrEP, which built on strong condom promotion, and test and treat programs. However, the HIV-negative partners may still be at risk of HIV transmission when they have sexual partners outside their relationship. The development of varied interventions by and with communities, tailored to their local contexts, may be required to support safe behaviours outside the relationship to minimise the risk for HIV transmission.

## Data Availability

A Research Data Management Plan has been developed for this research. All data is held within a university data repository to ensure transparency.

## Code Availability (Software Application or Custom Code)

Analysis was undertaken via StataIC 15 (64-bit).
